# Erbium-Based Perfusion Contrast Agent for Small-Animal Microvessel Imaging

**DOI:** 10.1155/2017/7368384

**Published:** 2017-11-15

**Authors:** Justin J. Tse, P. Joy Dunmore-Buyze, Maria Drangova, David W. Holdsworth

**Affiliations:** ^1^Imaging Research Laboratories, Robarts Research Institute, Western University, London, ON, Canada N6A 5B7; ^2^Department of Medical Biophysics, Western University, London, ON, Canada N6A 5C1; ^3^Department of Medical Imaging, Western University, London, ON, Canada N6A 5B7; ^4^Department of Surgery, Western University, London, ON, Canada N6A 5B7

## Abstract

Micro-computed tomography (micro-CT) facilitates the visualization and quantification of contrast-enhanced microvessels within intact tissue specimens, but conventional preclinical vascular contrast agents may be inadequate near dense tissue (such as bone). Typical lead-based contrast agents do not exhibit optimal X-ray absorption properties when used with X-ray tube potentials below 90 kilo-electron volts (keV). We have developed a high-atomic number lanthanide (erbium) contrast agent, with a K-edge at 57.5 keV. This approach optimizes X-ray absorption in the output spectral band of conventional microfocal spot X-ray tubes. Erbium oxide nanoparticles (nominal diameter < 50 nm) suspended in a two-part silicone elastomer produce a perfusable fluid with viscosity of 19.2 mPa-s. Ultrasonic cavitation was used to reduce aggregate sizes to <70 nm. Postmortem intact mice were perfused to investigate the efficacy of contrast agent. The observed vessel contrast was >4000 Hounsfield units, and perfusion of vessels < 10 *μ*m in diameter was demonstrated in kidney glomeruli. The described new contrast agent facilitated the visualization and quantification of vessel density and microarchitecture, even adjacent to dense bone. Erbium's K-edge makes this contrast agent ideally suited for both single- and dual-energy micro-CT, expanding potential preclinical research applications in models of musculoskeletal, oncological, cardiovascular, and neurovascular diseases.

## 1. Introduction

It is increasingly important in preclinical research to study the vasculature in both soft tissue and bone [1–5]. This includes visualization, quantification, and characterization of microvessels (i.e., vessels less than 10 *μ*m in diameter). Micro-computed tomography (micro-CT) can provide images with spatial resolution better than 5 *μ*m in both intact specimens and in* ex vivo* small animals [6–8]. However, blood-filled vessels lack inherent radiographic contrast, requiring the use of an exogenous contrast agent that can pass through capillaries and be retained within the vascular system. The increased contrast provided by such an agent facilitates automated (or semi-automated) segmentation (i.e., separation) of the perfused vasculature from surrounding tissues.

The performance (sensitivity, specificity, and accuracy) of vessel segmentation algorithms has been shown to be dependent on the signal-to-noise ratio (SNR, defined as the ratio of the vessel signal to background noise) between the contrast-enhanced vasculature and surrounding tissue in the micro-CT image [9]. Higher SNR results in a more robust and objective classification of the perfused vessels, leading to a more accurate assessment of their microarchitecture. The SNR can be increased by either reducing background noise or increasing the signal intensity within the vessel. However, decreasing micro-CT image noise is typically impractical, as it is achieved through much longer scan times [10]. Therefore, the most effective method to increase vessel SNR is to increase the contrast within the vasculature, using a radiopaque exogenous contrast agent.

Several formulations of exogenous vascular contrast agents are commonly available. Most clinical contrast agents for* in vivo* use are iodine-based; however, their small molecular size of <800 Da results in rapid clearance (i.e., within minutes) via the kidneys [11, 12]. Even in postmortem studies with increased iodine concentrations and scan times, the short retention time of these iodine-based agents makes them unsuitable for microvessel studies. Preclinical exogenous agents, of larger molecular sizes (i.e., >1100 Da), can remain within the blood pool for hours [13, 14]. These contrast agents are typically iodine-, barium-, or lead-based and have been used effectively to study vessel microarchitecture in the heart [15, 16], kidney [17, 18], tumours [19, 20], nerves [21–23], and long bones [24, 25]. However, these preclinical contrast agents do not exhibit optimal X-ray absorption (and hence do not optimize SNR) on a large installed base of micro-CT machines that typically operate at a maximum of 90 kilo-electron volts (keV).

X-ray absorption, responsible for observed contrast within X-ray images, is influenced by the K-edge of the contrast material (i.e., the energy required to eject an inner K-shell electron). The K-edges for common preclinical contrast agents are 33 keV for iodine, 37.4 keV for barium, and 88 keV for lead. These K-edge energies are not optimally matched for typical micro-focus tubes operating at a peak potential of 90 kVp, as the K-edge energies are located either at the low- or high-energy range of the output spectrum of the tube. Ideally, a contrast agent with a K-edge closer to the mean energy of the output spectrum of these micro-CT machines (i.e., ~50 keV) would provide enhanced X-ray absorption.

The lanthanide erbium (Er), with a K-edge at 57.5 keV, would provide the contrast necessary for micro-CT scanners operating at 90 kVp. An Er-based contrast agent would also provide an additional benefit for dual-energy micro-CT studies, which require CT scans above and below the K-edge of the material of interest [26]. In this study, we describe a novel contrast agent based on erbium oxide (Er_2_O_3_) nanoparticles (nominal diameter of ~50 nm) and illustrate a process by which Er_2_O_3_ nanoparticles form a colloidal suspension in a continuous-phase fluid (i.e., two-part liquid silicone elastomer). This methodology of contrast agent fabrication resulted in a high-atomic number (and consequently highly X-ray attenuating)* ex vivo* vascular perfusion contrast agent, with sufficiently low viscosity (19.2 mPa·s) to ensure the perfusion of the microvascular network (<10 *μ*m).

Using micro-CT, we demonstrate the efficacy of the custom contrast agent in a postmortem murine model. The contrast agent perfused the smallest vessels (i.e., capillaries) and provided increased SNR, facilitating the visualization of microvessels with diameter < 10 *μ*m. The Er-based contrast agent provided a greater SNR than commercially available agents, while also possessing a more appropriate absorption K-edge energy (57.5 keV). The resulting increase in vessel contrast would enhance the performance and automation of segmentation algorithms in all types of vascular networks and small-animal models (for both single- and dual-energy studies). This approach will be applicable in many preclinical studies, including musculoskeletal, cardiovascular, neurovascular, and oncological research programs.

## 2. Materials and Methods

### 2.1. Er-Based Contrast Agent Preparation

Erbium oxide (Er_2_O_3_) nanoparticles (NPs) were chosen as the main constituent of the contrast agent, due to their high X-ray attenuation and availability in a nanoparticulate powder (nominal diameter ~ 50 nm). To deliver the Er_2_O_3_ NPs throughout the vascular network, a commercially available two-part silicone elastomer (commonly used for vascular perfusion, Microfil MV-132, Flowtech Inc., Carver, MA, USA) was chosen as the carrier matrix. Uncured, this silicone elastomer has a manufacturer-reported viscosity of 20 mPa·s and when cured, the silicone matrix entrains the suspended Er_2_O_3_ NPs to form a stable silicone cast of the perfused vasculature. Initial experiments revealed difficulties incorporating the Er_2_O_3_ NPs within the two-part silicone elastomer. Analysis, via confocal fluorescence microscopy, of the uncoated Er_2_O_3_ powder revealed the tendency of the NPs to naturally aggregate into clusters > 1 *μ*m, due to van der Waals forces—nanoparticulate powders have been shown to naturally clump and form much larger particle sizes when left uncoated or untreated [27, 28]. Large aggregates such as these could clump together and prevent the perfusion of arterioles and capillaries, inhibiting perfusion of the venous system. Thus, to address the fact that uncoated nanoparticles tend to aggregate into large clumps (i.e., >100 *μ*m) a method was devised to ensure the size of the Er_2_O_3_ NPs in the final product remained smaller than 100 nm, as follows.

#### 2.1.1. Er_2_O_3_ NP Silicone Elastomer Suspension

Uncoated Er_2_O_3_ NPs of ~50 nm nominal diameter (Nanostructured and Amorphous Materials, Houston, TX, USA) were ground using a mortar and pestle for ~5 minutes to break down large aggregates. The ground powder was then mixed with an additive-free clear two-part silicone elastomer (Microfil MV-132, Carver, MA, USA), which is mixed in a ratio of two-part MV-Diluent to one-part MV-132. To prepare 30 mL of contrast agent, 4.0 g of ground Er_2_O_3_ (i.e., 13.3% w/v) was added to 17.47 mL of MV-Diluent and 8.73 mL of MV-132; the remaining 3.8 mL was comprised of the curing agent described below, which was added immediately prior to perfusion into the animal. The uncured suspension of Er_2_O_3_ powder and silicone elastomer was probe sonicated (Branson Digital Sonifier 450D, standard 13 mm tapped horn, Crystal Electronics, Newmarket, ON, Canada) for a total of 35 minutes with 25% amplitude and a duty cycle of 30 s ON to 10 s OFF. Due to the intense heat generated during sonication, the samples were immersed in an ice bath and sonication was performed in three intervals interspersed with 5–10-minute cool-down periods. The Er_2_O_3_ NP silicone elastomer suspension was prepared several hours prior to perfusion, to allow particle aggregates to settle, and then decanted prior to use. If prepared further in advance, sonication of the suspension of 5–10 minutes is required to ensure particle resuspension.

#### 2.1.2. Curing Agent

To facilitate consistent and controlled curing of the Er_2_O_3_ NP silicone elastomer suspension, a tin-based curing agent was prepared in-house. The curing agent comprised a solution of 40% (w/w) dibutyltin dilaurate (Sigma Aldrich, St. Louis, MI, USA) in tetraethyl orthosilicate (Sigma Aldrich, St. Louis, MI, USA), which was mixed using a magnetic stirrer for several hours until it became a homogeneous pale-yellow transparent solution.

### 2.2. Er-Based Contrast Agent Characterization

Particle and aggregate sizes of the “raw” and sonicated Er_2_O_3_ powder were evaluated visually using confocal fluorescence microscopy. Prior to sonication, drops of raw Er_2_O_3_ powder mixed within the two-part silicone elastomer were dispensed on a glass bottom microwell dish (MatTek Corporation, Ashland, MA, USA). Following sonication, drops of the prepared Er-based suspension were placed on a separate microwell dish. Samples were analyzed using confocal fluorescence microscopy (Leica DMi8, Wetzlar, Germany), an Ar 488 nm laser for excitation, and emission bandwidths of 493–739 nm. To visualize particle sizes within the nonsonicated “raw” sample and sonicated suspension, a 20x (HC PL APO CS2 20x/0.75 DRY) and 63x (HC PL APO CS2 63x/1.40 OIL) objective lens were used, respectively.

Dynamic light scattering (DLS) was used to quantify the size distribution of the prepared Er-based suspension. A 10% (v/v) dilution of the suspension in MV-Diluent was prepared and analyzed with DLS (ZetaSizer Nano Instrument, Malvern Instruments Ltd., Malvern, UK). Measurements were performed at room temperature (25°C) in a quartz cuvette (1 mg/mL).

The viscosity of the contrast agent was measured using a lab-based Modular Compact Rheometer (MCR 302, Anton Paar, Graz, Austria); the measured viscosity was used to correct the DLS measurements.

### 2.3. Animals

All animal studies were approved by the Animal Use Subcommittee at Western University (protocol #2015-018). Five male C57BL/6 mice (~30 g) were used for this study. The mouse model was selected to demonstrate the capability of the Er_2_O_3_ contrast agent to perfuse the microvasculature of the smallest of the commonly used small-animal models. Anesthetized mice were first exsanguinated with sterile saline followed by perfusion with the Er contrast agent. To prevent blood clot formation during exsanguination, sterile 0.9% (w/v) saline was heparinized to 0.4% (1 mL of heparin (Sandoz, QC, Canada) in 250 mL saline). Sterile tubing (Baxter Canada, Mississauga, ON, Canada), 1.8 m in length, was used to connect the saline bag to a blunted 21 G × 3/4′′ butterfly catheter (BD, Franklin Lakes, NJ, USA). The saline IV bag was hung 127 cm above the surgery table, thereby providing a pressure of 94 mmHg. Five minutes prior to the start of the procedure the mice were given a 100 *μ*l intraperitoneal injection of heparin. After induction of anesthesia (3% isoflurane (Baxter Canada, Mississauga, ON, Canada) in O_2_ at a rate of 2 mL/min) an incision was made along the thoracic cavity exposing the heart. The butterfly catheter was carefully inserted into the left ventricle parallel to the septum. A drop of cyanoacrylate (Krazy Glue, Elmer's Products, Atlanta, GA, USA) was applied at the entry point of the catheter into the left ventricle to avoid accidental piercing of the septum. The right atrium was clipped to allow for circulatory system drainage. The heparinized saline solution was perfused throughout the circulatory system for 10 minutes to ensure complete removal of the blood.

During saline perfusion, 3.8 mL (12.7% v/v) of curing agent was added to 36.2 ml Er_2_O_3_-based silicone elastomer suspension and vortexed (VWR® Fixed Speed Vortex Mixer, Radnor, PA, USA) continuously for 8 minutes. The contrast agent was injected into an empty IV bag (with separate 1.8 m of surgical tubing) and hung 160 cm above the mice (129 mm Hg). While this value is greater than the mean arterial pressure (MAP) of mice (~103 mm Hg) [29, 30], it was chosen to ensure complete perfusion of the animal before the contrast agent cured. Furthermore, the perfusion pressure used in this study is significantly lower than the >150 mmHg used in prior studies using the lead-based Microfil agent [22, 31–33]. Perfusion at a pressure more closely matched to peak systolic pressure (i.e., ~120 mmHg) reduces the risk of vascular dilation and capillary rupture. The contrast agent was let to freely perfuse through the animal until completely cured, which occurred approximately ~35 minutes after start of perfusion. Following contrast agent curing, mice were placed in 10% neutral buffered formalin overnight, prior to micro-CT scanning.

### 2.4. Data Collection and Analysis

Whole body mouse scans were acquired with a preclinical micro-CT scanner (Vision 120, GE Healthcare, London, ON, Canada). The scan parameters were 90 kVp (no added filtration), 40 mA, 900 views, 0.4° increment angle over 360°, geometric magnification of 1.13, and 16 ms exposure, resulting in a total exposure time of 14.4 s and 576 mAs. Including the time required for gantry motion and recording of image projections, the total acquisition time was 5 minutes. The projection images were binned 2 × 2 prior to reconstruction for a final isotropic voxel spacing of 100 *μ*m.

Higher resolution scans of the hindlimb regions were acquired on a specimen scanner (Locus, GE Healthcare, London, ON, Canada) using a 3 hr scan protocol (900 views, 80 kVp, 80 *μ*A, no added filtration, 0.4° increment angle over 360°, geometric magnification of 1.41, 15-frame averaging, and 2 × 2 binning for a final isotropic voxel spacing of 40 *μ*m). To prevent sample motion during these high-resolution image acquisitions, the perfused mouse was placed in a 50 mL tube.

Confirmation of perfusion of microvessels (i.e., <10 *μ*m) was achieved by high-resolution micro-CT. Fabricated microvessel constructs or synthetic capillaries have been utilized to evaluate microvessel perfusions in the past [34, 35], but fabricating synthetic vessels with diameters on the order of 10 *μ*m remains technically challenging. Fortunately, the mouse kidney is a well-characterized organ system, with known vessel diameters ranging from the renal artery (~0.3 mm) to capillaries (~10 *μ*m) [36, 37]. The kidney contains many glomeruli (responsible for the waste removal and blood filtering), which are comprised of capillaries in a bundle of ~75 *μ*m diameter [38]. Therefore, an excised Er_2_O_3_-perfused kidney was embedded in paraffin in a 1.2 mL tube (Corning®, Corning, NY, USA) and scanned with a specimen scanner (Locus SP, GE Healthcare, London, ON, Canada), using a 16 hr protocol. Scan parameters were 80 kVp, 80 *μ*A, 900 projections, no added filtration, 0.4° increment angle over 360°, geometric magnification of 3.83, 14-frame averaging, and 1 × 1 binning for a final isotropic spatial resolution of 4.8 *μ*m.

The micro-CT scanners used in this study were all equipped with a CsI-based energy-weighted detector. It has been shown that the peak response of these detectors [39] is very close to the absorption K-edge of Er (57.5 keV), making them ideally suited for detection of an erbium-based contrast agent.

Each of the scans contained calibrators of water and air, which were used for image calibration and conversion into Hounsfield units (HU). This allowed us to quantify the amount of contrast enhancement of perfused vasculature, based on the the CT signal level in HU within various organs throughout the vascular system. Using 3D visualization and analysis software (MicroView, GE Healthcare, London, ON, Canada), regions of interest (ROI) 500 × 500 × 500 *μ*m were generated in each region and the mean HU values recorded. Specifically, for all mice, the mean HU was determined from the heart (left ventricle), testes, and inferior vena cava (IVC), as they represented the beginning, middle, and end of the perfusion pathway, respectively. The CT signal levels within the selected organs were compared to cortical bone within the diaphysis region (i.e., the densest endogenous contrast) and a commercially available lead-based contrast agent. A rat hindlimb previously perfused with the widely used and commercially available lead-based contrast agent (Microfil MV-122, Flowtech Inc., Caver, MA, USA) was scanned using the 100 *μ*m acquisition protocol.

All statistical analyses were performed using Prism 6 (GraphPad Software Inc., La Jolla, CA, USA). Repeated measures ANOVA was used to test for statistical differences between all Er-based contrast-enhanced regions (i.e., heart, testes, and IVC) and cortical bone. In a separate test, one-way unpaired ANOVA was performed to compare the mean attenuation in Er-perfused vessels against cortical bone and the Microfil MV122-perfused rat femoral artery. Statistical differences were noted if a *p* < 0.05 was achieved.

## 3. Results

### 3.1. Efficacy of an* Ex Vivo* Er-Based Contrast Agent for Vascular Perfusion

An effective preclinical postmortem X-ray compatible vascular contrast agent must be comprised of small, X-ray attenuating particles homogeneously suspended within a low viscosity medium. These characteristics will ensure uniform contrast enhancement of perfused vasculature, including microvessels with diameter < 10 *μ*m (i.e., capillaries). Automated segmentation algorithms, which are typically based on grey-scale levels, require homogeneous perfusion of microvessels to effectively separate perfused vasculature from surrounding tissues, so it is essential to employ an appropriate particle size, uniformly distributed in the carrier medium.

**Figure 1:**
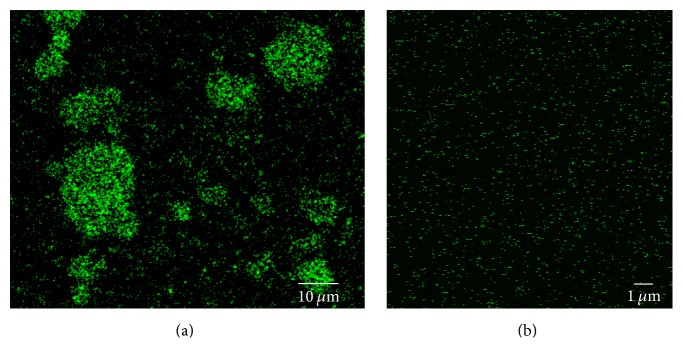
Confocal fluorescence microscopy images of (a) nonsonicated raw Er_2_O_3_ powder naturally aggregated to large microsized (>10 *µ*m) particles when mixed within the two-part silicone elastomer, making the suspension not suitable for microvascular perfusion. However, with sonication, nanosized (~70 nm) particles were achieved (b).

Ultrasonic cavitation (sonication) was used to successfully break up large aggregates of Er_2_O_3_ to nm-sized aggregates, which could be homogeneously incorporated within the two-part silicone matrix. Following intense sonication, a visually homogeneous suspension of 13.3% w/v Er_2_O_3_ within the two-part silicone elastomer was achieved. The Er_2_O_3_ NPs were found to remain in suspension for several days, allowing for the contrast agent to be prepared several days prior to use. Confocal fluorescence microscopy visually confirmed that the size of the sonicated nanoparticles within the Er_2_O_3_ contrast agent suspension (Figure 1(b)) was less than 100 nm—a size that can pass easily through the microvessels of any vascular system.

The ability of a contrast agent to perfuse the microvasculature also depends on its viscosity. Measuring the viscosity of the uncured Er-based contrast agent—at 19.2 mPa·s—demonstrated agreement with the 20 mPa·s viscosity reported by the manufacturer of the two-part silicone elastomer, confirming that the uncured Er_2_O_3_ contrast agent is able to pass through small vessels under standard perfusion pressures.

**Figure 2:**
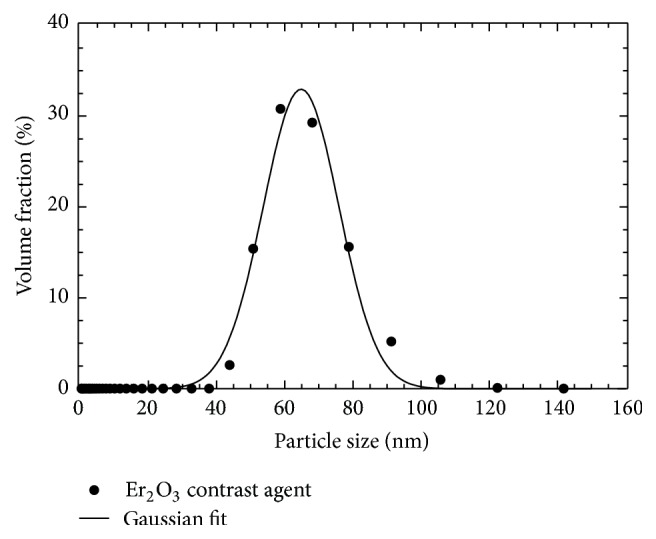
Dynamic light scattering (DLS) results demonstrating the particle size distribution of a sample of the Er_2_O_3_ contrast agent. Average particle size is 72.2 ± 2.2 nm. Results of a suspension that was mixed and subsequently stored for 2 years are shown in S1.

Based on the measured viscosity of 19.2 mPa·s, the DLS measurement reported a Gaussian particle size distribution with mean hydrodynamic diameter of 64.8 nm, standard deviation of 11.1 nm, and a range from 44 to 122 nm (Figures 2 and S1; see Supplementary Material available online at https://doi.org/10.1155/2017/7368384). Measurements of particle size and carrier viscosity indicated that the prepared Er_2_O_3_ suspension should easily pass through microvessels; this aspect of performance was further evaluated by micro-CT imaging of intact perfused mice.

**Figure 3:**
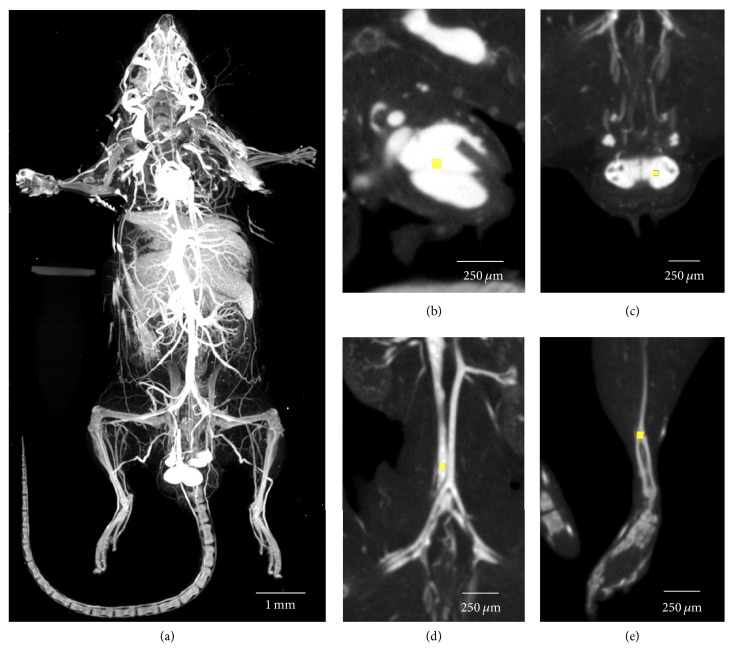
Rebinned 100 *µ*m voxel images where the (a) maximum intensity projection (MIP) of a whole body perfused mouse demonstrates that the attenuation of the Er_2_O_3_ contrast agent in the vasculature is higher than the mouse's skeletal structure. Quantitative measurements of attenuation (in HU) were obtained from regions drawn within heart (b), testes (c), inferior vena cava (d), and cortical bone (e).

Whole Er_2_O_3_-perfused C57Bl/6 mice scanned with 50 *μ*m isotropic voxel spacing, and rebinned 2 × 2 to a final resolution of 100 *μ*m, revealed a uniform and homogeneous distribution of the cured Er_2_O_3_ contrast agent within the vasculature throughout the entire perfused mouse (Figure 3). The vasculature displayed enhanced contrast in comparison to surrounding tissues throughout an intact animal; importantly the attenuation of the contrasted vessels was higher than that of bone.

**Figure 4:**
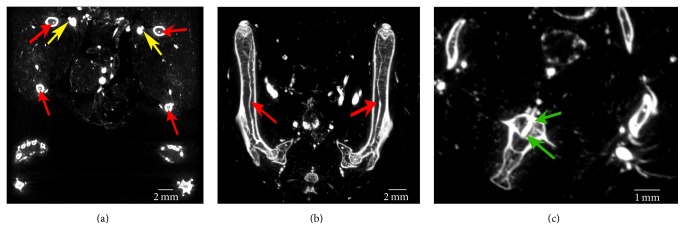
Multiplanar reformatted images at 40 *µ*m, resulting from 2 × 2 rebinning of 20 *µ*m acquired micro-CT scans, clearly depict the ability to visualize the extent of the nutrient arteries, which run along the tibia and femur. Red arrows highlight the nutrient arteries in cross-section in (a) and along their entire length in (b). At this resolution the depiction of parallel arteries and veins (yellow arrows) indicates successful perfusion through the capillary network. The ability to visualize vessels as they pass through a foramen (green arrows) into bone is depicted in (c).

Scans of Er contrast-perfused hindlimbs were acquired with 20 *μ*m isotropic voxel spacing and subsequently rebinned 2 × 2 for a final resolution of 40 *μ*m (Figure 4), to observe the smaller vasculature next to the dense bony structures of the femur and tibia. From these results, we were able to clearly see a feeding artery that runs within (Figure 4(a)) and along (Figure 4(b)) the entire length of each long bone. At this higher resolution, smaller structures such as a foramen (i.e., an opening for blood vessels to enter bone) can be visualized (Figure 4(c)). The ability to differentiate the foramen from the vessel running through it is particularly noteworthy, as this is not possible with other contrast agents that have lower attenuation coefficients. The observed perfusion of the venous system (Figure 4(a) yellow arrows) suggests successful perfusion of the capillaries, which is further supported by the lack of visible contrast agent pooling within the interstitial space (pooling might have been observed if overpressurization during perfusion had caused microvessel rupture).

### 3.2. Visualization of Capillary Bed Perfusion

**Figure 5:**
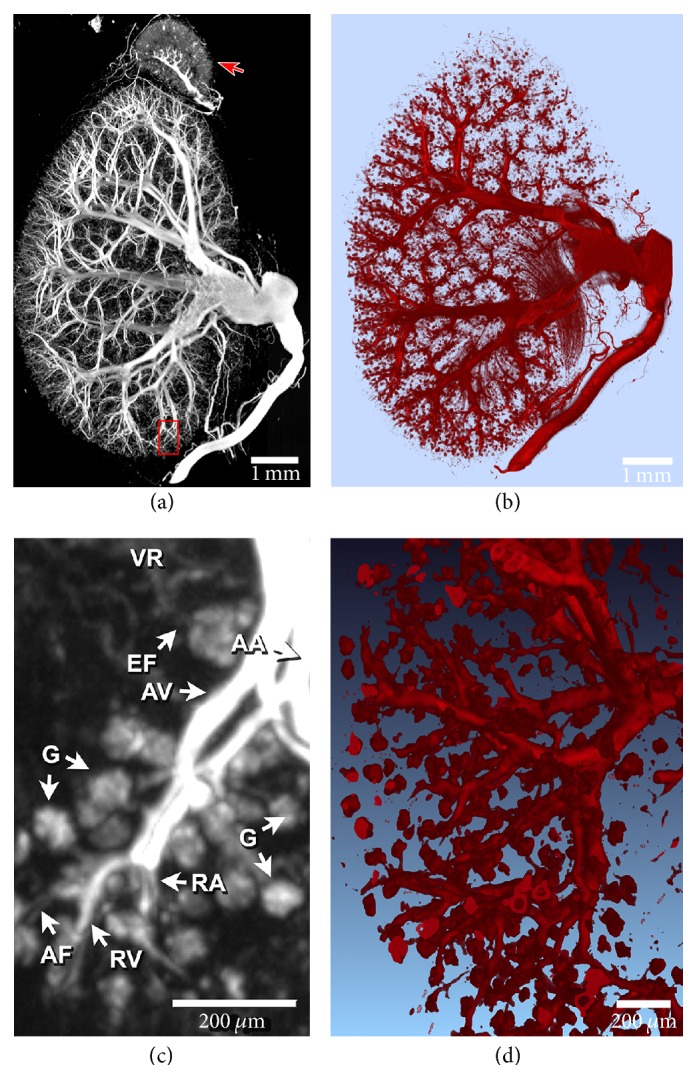
(a) MIP of an entire mouse kidney and attached adrenal gland (arrow) perfused with the new Er contrast agent. (b) 3D rendering of the perfused kidney with a plane cut to demonstrate an entire intact vascular tree. (c) Magnified 0.35 mm thick slice MIP of the area outlined in red in (a), demonstrating 6-7th level arterial branching. (d) 3D rendering of the terminal arteriole branches, ending in the glomeruli (the kidney's spherical capillary bed). G: glomeruli; AF: afferent arteriole; EF: efferent arteriole; RA: cruciate radial artery; RV: cruciate radial vein; AA: arcuate artery; AV: arcuate vein; and VR: vasa recta.

The results of the high-resolution micro-CT scan revealed that the Er_2_O_3_ contrast agent successfully perfused the entire continuous, well-ordered vascular tree of the kidney (Figure 5). The contrast enhancement of the vasculature was sufficiently high, such that a single grey-scale threshold separated perfused vasculature from surrounding tissues, allowing for the generation of 3D images of the kidney vessel microarchitecture (Figure 5(b)). The virtual Er_2_O_3_ vascular “cast” (Figures 5(a) and 5(b)) showed complete perfusion from the abdominal aorta (i.e., the main feeding vessel) down to the 6th and 7th arterial branches (i.e., glomeruli afferent arterioles). Previous research has shown that mouse glomeruli afferent arterioles can be as small as ~13 *μ*m [36]; thus, visualization of individual glomeruli (i.e., several capillaries) suggests that our contrast agent is able to perfuse structures < 13 *μ*m (Figures 5(c) and 5(d)). While previous research has shown perfusion of the kidney down to the afferent arterioles [36, 40–42] using a commercially available lead-based contrast agent, the main advantage of the Er_2_O_3_-based contrast agent is its ability to provide higher contrast and SNR.

### 3.3. Contrast Enhancement Provided by the Er-Based Contrast Agent in Micro-CT

**Figure 6:**
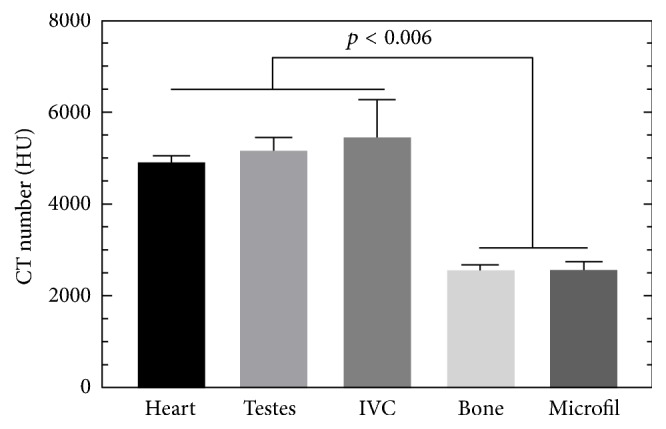
Heart, testes, and inferior vena cava (IVC) were chosen to represent the start, middle, and end of the perfusion route. The attenuation (HU) of the Er_2_O_3_-based contrast agent in all three regions was significantly higher than that of cortical bone (*p* < 0.006, repeated measures ANOVA). Importantly, the attenuation of the Er_2_O_3_-enhanced vasculature was significantly higher than that commercially available lead-based Microfil MV122 (one-way ANOVA, *p* < 0.0001), while there was no difference between Microfil MV122 and cortical bone (*p* > 0.9999).

The measured mean CT values for the heart (4094 ± 264 HU), testes (4107 ± 182 HU), and IVC (4001 ± 305 HU), compared in Figure 6, showed no significance difference (*p* = 0.3940) between these three regions. On the other hand, the mean signal from cortical bone (2359 ± 207 HU) and the lead-based contrast agent Microfil MV-122 (2683 ± 77.6 HU) were significantly lower than the signal from the Er contrast-agent perfused vasculature (*p* < 0.006 and *p* < 0.0001 for bone and Microfil, respectively). The approximately 1400 HU difference in signal between that provided by the Er_2_O_3_-based contrast agent and cortical bone will aid in facilitating the automatic segmentation of vessels from surrounding bone, which is not possible when commercially available contrast agents, such as Microfil MV 122, are used.

## 4. Discussion

We have demonstrated a methodology for the homogeneous incorporation of Er_2_O_3_ nanoparticles within a two-part silicone elastomer, forming a colloidal suspension capable of providing high X-ray attenuation that can facilitate the visualization and characterization of microvessels within a small-animal model. In this study, we characterized and investigated the capabilities of the custom* ex vivo* Er-based vascular perfusion contrast agent.

Ultrasonic cavitation successfully broke down large naturally occurring Er_2_O_3_ aggregates (i.e., >100 *μ*m) into nm-sized particles (Figures 1(b) and 2) suspended within a silicone carrier. The prepared suspension was determined to possess low viscosity and a narrow particle size distribution that would facilitate the perfusion of intact whole body mice (Figure 3). Micro-CT scans acquired with both 50 and 20 *μ*m isotropic voxel spacings revealed whole-mouse perfusion and higher-ordered vascular branching (i.e., 1st to 3rd order) and visualization of vessels within bone (Figures 3 and 4). High-resolution scans with 5 *μ*m spatial resolution demonstrated well-characterized vascular microarchitecture within a perfused kidney, with observed vascular filling down to vessels <13 *μ*m in diameter and contrast enhancement of capillary beds (i.e., glomeruli, Figure 5). Additionally, the attenuation of the Er-based contrast agent was found to be significantly higher than that of cortical bone (i.e., the densest naturally occurring substance within our samples) and the commonly used lead-based Microfil (MV-122) vascular contrast agent (Figure 6). This study clearly demonstrates the efficacy of the custom Er-based suspension as an* ex vivo* micro-CT vascular perfusion contrast agent.

An important benefit of the presented Er-based contrast agent is an X-ray attenuation coefficient that is significantly higher than that of both bone and other existing contrast agents. This difference facilitates the separation of microvessels from both soft tissue and bone in the images and can also result in a reduction of scan time. While shorter scans result in a greater amount of noise [10], the higher contrast between the Er contrast agent within the vasculature and surrounding tissue ensures that SNR remains high despite the shorter scan times. The approach used to incorporate the Er nanoparticles within the suspension (i.e., ultrasonic cavitation) renders the approach amenable to the production of custom contrast agents of varying elemental compositions and concentrations. Furthermore, silicone-compatible colorants can also be introduced within the silicone media to allow for the customization of the contrast agents' visual appearance against tissue; this may be useful for macroscopic visualization and postmortem histological analysis.

In the current implementation, each working volume of contrast agent (i.e., 30 ml) is prepared individually, requiring approximately one hour of operator time. Larger volumes of contrast agent could be prepared in advance, with the curing catalyst being added just prior to usage. In this case, additional sonication may be required to ensure resuspension of aggregated particles (Figure S1).

As with other cast-forming contrast agents (e.g., Microfil) the new Er-based contrast agent is limited to applications of postmortem vascular analysis at study endpoints. This limitation requires that larger cohorts of animals are needed to assess changes to the vasculature over periods of time. An* in vivo* contrast agent would make investigations with reduced sample size possible and allow for the study of vascular changes within the same animal over time; however, development of an* in vivo* contrast agent is not within the scope of this study. Currently, there exist* in vivo* contrast agents that reside within the blood pool for extended periods of time [43, 44]; thus, we expect that the incorporation of Er into an* in vivo* agent is possible.

## 5. Conclusions

We have demonstrated the effectiveness of an Er-based suspension as a single-energy X-ray vascular contrast agent that significantly enhances the contrast—in comparison to surrounding dense bone and commercially available lead-based contrast agents (Figure 6)—of perfused vasculature within small animals (Figures 3–5). With an absorption K-edge at 57.5 keV, the Er-based contrast agent is also ideally suited for dual-energy micro-computed tomography (DECT) on a large installed base of high-resolution preclinical micro-CT machines that operate at up to 90 kVp. The combination of the Er_2_O_3_-based vascular perfusion contrast agent with optimized DECT scan protocols and spectral shaping (using X-ray filtration) would facilitate rapid and automatic quantitative segmentation of perfused vasculature from surrounding tissues [26], a process that is otherwise difficult due to partial volume effects that can limit traditional single-energy CT scans. Dual-energy CT-based material separation has been shown to be beneficial in studying a range of diseases in clinical applications (i.e., gout, cardiovascular, and cancer) [45–47], by allowing for the quantitative separation of the material of interest from surrounding tissues. The novel contrast agent that we describe has the potential to provide these advantages of DECT-based quantification and segmentation for preclinical investigations of vascular changes in small-animal models.

## Supplementary Material

Figure S1. DLS measurements of a two-year old Er-based suspension. Presented are the particle size distributions after the sample had been re-sonicated for either 5 or 10 minutes.

## Data Access

All data needed to evaluate the conclusions in the paper are present in the paper and/or the Supplementary Materials. Additional data related to this paper may be requested from the authors.
