# Corrigendum to “Erbium-Based Perfusion Contrast Agent for Small-Animal Microvessel Imaging”

**DOI:** 10.1155/2018/2165693

**Published:** 2018-08-23

**Authors:** Justin J. Tse, P. Joy Dunmore-Buyze, Maria Drangova, David W. Holdsworth

**Affiliations:** ^1^Imaging Research Laboratories, Robarts Research Institute, Western University, London, ON, Canada N6A 5B7; ^2^Department of Medical Biophysics, Western University, London, ON, Canada N6A 5C1; ^3^Department of Medical Imaging, Western University, London, ON, Canada N6A 5B7; ^4^Department of Surgery, Western University, London, ON, Canada N6A 5B7

In the article titled “Erbium-Based Perfusion Contrast Agent for Small-Animal Microvessel Imaging” [1], there were errors in the scale bars in Figure 3, which should be corrected as follows:

## Figures and Tables

**Figure 3:**
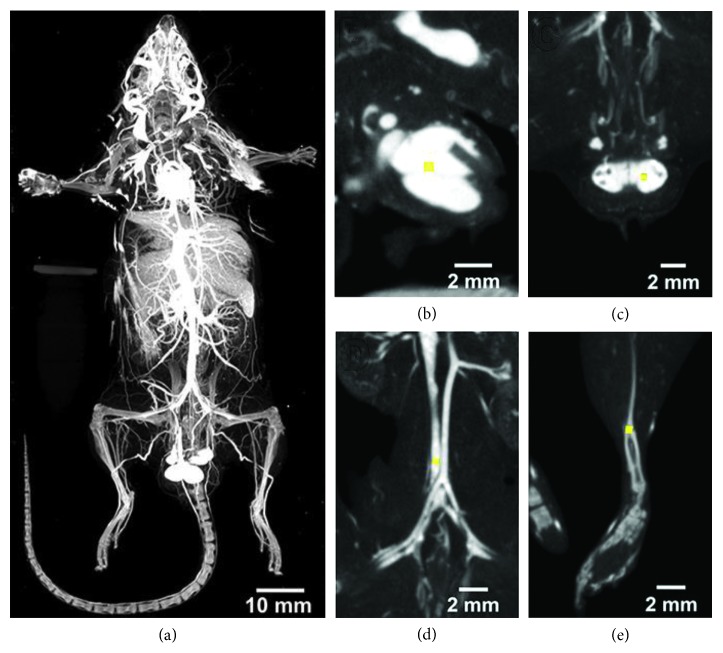
Rebinned 100 *μ*m voxel images where the (a) maximum intensity projection (MIP) of a whole body perfused mouse demonstrates that the attenuation of the Er2O3 contrast agent in the vasculature is higher than the mouse's skeletal structure. Quantitative measurements of attenuation (in HU) were obtained from regions drawn within heart (b), testes (c), inferior vena cava (d), and cortical bone (e).
